# Genome Target Evaluator (GTEvaluator): A workflow exploiting genome dataset to measure the sensitivity and specificity of genetic markers

**DOI:** 10.1371/journal.pone.0182082

**Published:** 2017-07-27

**Authors:** Arnaud Felten, Laurent Guillier, Nicolas Radomski, Michel-Yves Mistou, Renaud Lailler, Sabrina Cadel-Six

**Affiliations:** Université PARIS-EST, ANSES, Laboratory for Food Safety, Maisons-Alfort, France; Animal and Plant Health Agency, UNITED KINGDOM

## Abstract

Most of the bacterial typing methods used to discriminate isolates in medical or food safety microbiology are based on genetic markers used as targets in PCR or hybridization experiments. These DNA typing methods are important tools for studying prevalence and epidemiology, for conducting surveillance, investigations and control of biological hazard sources. In that perspective, it is crucial to insure that the chosen genetic markers have the greatest specificity and sensitivity. The wealth of whole-genome sequences available for many bacterial species offers the opportunity to evaluate the performance of these genetic markers. In the present study, we have developed GTEvaluator, a bioinformatics workflow which ranks genetic markers depending on their sensitivity and specificity towards groups of well-defined genomes. GTEvaluator identifies the most performant genetic markers to target individuals among a population. The individuals (i.e. a group of genomes within a collection) are defined by any kind of particular phenotypic or biological properties inside a related population (i.e. collection of genomes). The performance of the genetic markers is computed by a distance value which takes into account both sensitivity and specificity. In this study we report two examples of GTEvaluator application. In the first example *Bacillus* phenotypic markers were evaluated for their capacity to distinguish *B*. *cereus* from *B*. *thuringiensis*. In the second experiment, GTEvaluator measured the performance of genetic markers dedicated to the molecular serotyping of *Salmonella enterica*. In one *in silico* experiment it was possible to test 64 markers onto 134 genomes corresponding to 14 different serotypes.

## Introduction

Genetic markers are important tools for biological systematics, epidemiological surveillance and investigations, or ecological genetics. By taking foodborne pathogens as example, the fact that some strains cause outbreaks and severe diseases, whereas others are only associated with mild symptoms in human, leads to define refined molecular targets according to these phenotypic sub-clusters. For example, seropathotypes of *Escherichia coli* have been proposed to identify most virulent strains in term of serotype associated with human epidemics, as well as hemolytic and uremic syndromes [1]. Thus, the epidemiological investigations of foodborne pathogens require accurate isolates typing methods beyond the species level. The recent development of whole genome sequencing (WGS) will impact the selection of specific and sensible targets to develop these innovative typing methods.

In the era of WGS, PCR-based typing approaches targeting small genomic regions keep their relevance by being extremely fast, cheap and with a great potential to be implemented with high-throughput equipment. However, assess appropriate genomic targets, both sensitive and specific, is an important issue. To meet this challenge, empiric approaches based on a limited quantity of genomic information and validated on a limited number of isolates are usually used. The tremendous increase of bacterial genomic information theoretically offers the opportunity to realize *in silico* analyses to evaluate the performance of the genomic targets selected [2]. Here, we have developed a bioinformatics tool called ‘Genome Target Evaluator’ (GTEvaluator), which makes it possible to rank the most suitable markers among a list, by calculating their sensitivity (S_e_) and specificity (S_p_) toward a genome dataset. A distance value is then computed by these two parameters (S_e_ and S_p_) providing a dataset-dependent estimation of the quality of the markers. A Bayesian stochastic approach was also set up to take into account the limited amount of information on which the calculations were performed (i.e. number of genomes). GTEvaluator provides a simple way to take rational decision on the choice of genetic markers by taking advantage of the large WGS resources now available.

In this study we have tested GTEvaluator on two well-known and widely distributed foodborne pathogens: *Bacillus* and *Salmonella*. The first test with *Bacillus* is used as a negative case-control. We wanted to differentiate, at the species level *B*. *cereus* and *B*. *thuringiensis*. We included in the input of the workflow, genomes of the two *Bacillus* species and genomic markers known to be common to both (*B*. *cereus* and *B*. *thuringiensis*) [3–10]. The second test with *Salmonella* is used as a case study. We wanted to differentiate several serotypes of *Salmonella enterica*. We established a list of genetic markers selected from several studies focusing on molecular serotyping of *S*. *enterica* serogroups and serotypes [11–16]. The performance of molecular markers was measured on a data set of 134 genomes corresponding to 14 serotypes. To design experimental situations which can be faced by biologists, the 14 serotypes were not equally represented in the genome dataset. GTEvaluator analysis provides an objective evaluation of the value of markers which takes into account the representativeness of the genomic dataset.

## Materials and methods

### Description of the workflow

**Fig 1:**
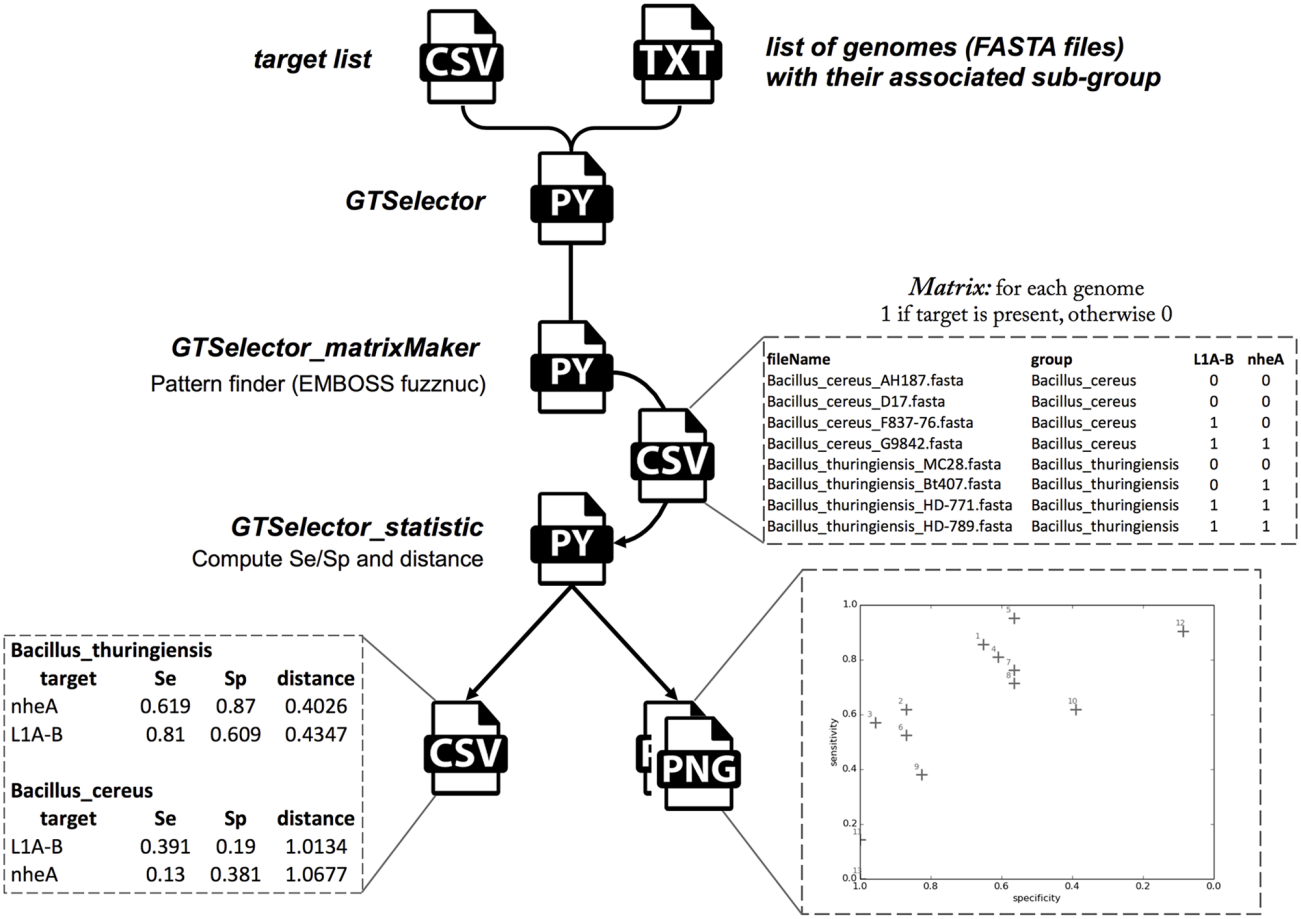
GTEvaluator workflow. The lists of genetic markers and genomes are the input files of a ‘GTEvaluator’ script which is based on the ‘fuzznuc’ pattern finder, and constituted of ‘GTEvaluator_matrixMaker’ and ‘GTEvaluator_statistic’ scripts for matrix file production (i.e. presence or absence of genomic markers for each genome) and statistical computation (i.e. specificity, sensitivity, statistical distances, and confidence intervals), respectively.

The ‘GTEvaluator’ workflow is composed of three Python scripts (Fig 1). The first script is called ‘GTEvaluator_matrixMaker’ and requires a list of genomes and a list of targets as inputs. This script uses ‘fuzznuc’ (version 6.6.0) to match all genetic markers on all genomes [17]. At this step, the user has the possibility to set two parameters: first, the number of 5’-nucleotides to be trimmed on primers if necessary and second, the maximal distance between forward and reverse primers. ‘GTEvaluator_matrixMaker’ produces a matrix file in tabular format reporting the ‘presence’ (1) or ‘absence’ (0) of the targets in the genomes. Presence is defined as a perfect match between the marker and the genomic target (100% of sequence homology). The ‘GTEvaluator_matrixMaker’ matrix is used as input for the second script ‘GTEvaluator_statistic’ which computes specificity, sensitivity, statistical distances (see below) and confidence intervals for each genetic marker and each pre-defined subgroup (i.e. species, serotypes, phenotypic classes, etc.).‘GTEvaluator_statistic’ generates a tabular file with the computed results for the markers which are sorted by growing statistical distances. If the number of subgroups does not exceed 6, a scatter plot is automatically generated displaying, for each group, the genomic markers as a collection of points whose positions are determined according to their sensitivity (vertical axis) and specificity (horizontal axis) (Fig 1).

The last script ‘GTEvaluator’ is a driving script which runs consecutively ‘GTEvaluator_matrixMaker’ and ‘GTEvaluator_statistic’.

### Statistical analysis

#### Evaluation of the performance of markers

Sensitivity (*S*_e_) and specificity (*S*_p_) were calculated for each marker associated to a group of genomes using the following formulas: $S_{e}=\frac{x_{11}}{x_{11}+x_{10}}$ and $S_{p}=\frac{x_{00}}{x_{00}+x_{01}}$(see Table 1). In the formulas, *x*_11_ and *x*_10_ represent the number of genomes in which genetic marker is present and absent respectively among the *n*_*1*_ genomes of the subgroup of interest *g*_1_; *x*_*00*_ and *x*_01_ represent the numbers of genomes, among the *n*_*0*_ genomes of group *g*_0,_ in which the genetic marker is absent and present, respectively. The *g*_0_ group is constituted by all the genomes not belonging to the *g*_1_ group.

**Table 1:** Typological variables describing the ‘presence’ (i.e. i = 1 or j = 1) and ‘absence’ (i.e. i = 0 or j = 0) of genetic markers (x_ij_) across subgroups of studied genomes (*g*). The genomes from the targeted subgroup and other subgroups are called *g*_1_ and *g*_0_, respectively.

	Genetic marker		Total
	Presence	Absence	
Genomes of the interest subgroup (*g*_1_)	x_11_	x_10_	n_1_
Other genomes from other subgroup(s) (*g*_0_)	x_01_	x_00_	n_0_

The different genetic markers were then ranked on the basis of their *d* value calculated as $d=\sqrt{{\left(1-S_{p}\right)}^{2}+{\left(1-S_{e}\right)}^{2}}$ that is a measure of the performance of markers taking into account both *S*_*e*_ and *S*_*p*_. The d value for a perfect genetic marker is zero. A non-specific or sensitive genetic marker for classifying groups would result in *d* = 1.

#### Uncertainty assessment

**Fig 2:**
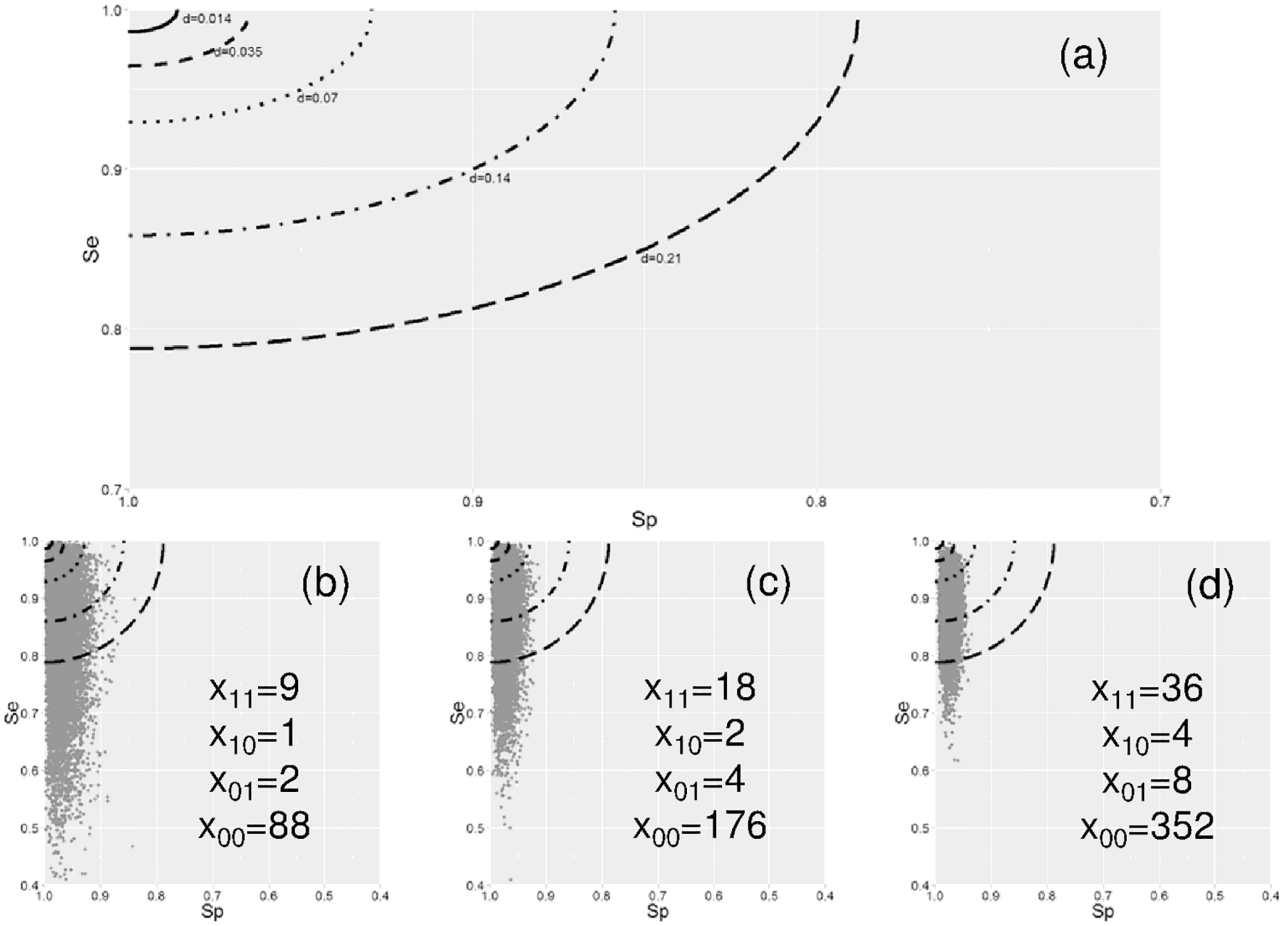
Simulated distances and uncertainties of specificity and sensibility implemented in GTEvaluator. A distance value (*d*) defines the performance of a marker in term of specificity (S_p_) and sensitivity (S_e_) across considered subgroups of genomes (Fig 2a). The uncertainty on specificity and sensitivity is presented for 100 (Fig 2b), 200 (Fig 2c), and 300 (Fig 2d) genomes in the dataset. A potential genomic marker of a given subgroup of genomes (x_ij_) is defined by his presence (i or j = 1) and absence (i or j = 0) in genomes of this subgroup (i) and others (j). Specificity and sensitivity are constant values (i.e. S_e_ = 0.900 and S_p_ = 0.977), and the targeted subgroup represents 20% of the genome dataset in the present simulation.

Bayesian confidence intervals of *S*_e_ and *S*_p_ were calculated with the following formula: *S*_*e*_|*a* ~ *beta*(*x*_11_ + 1, *n*_1_ − *x*_11_ + 1) and *S*_*p*_|*a* ~ *beta*(*x*_00_ + 1, *n*_0_ − *x*_00_ + 1) [18]. Beta distributions on *S*_e_ and *S*_p_ were used to compute uncertainty on *d* value (calculated as described above) by Monte-Carlo simulations [19]. The level of uncertainties on *d* was estimated by defining the number of simulations corresponding to coordinates {*S*_e_ = 0.990; *S*_p_ = 0.990} (d = 0.014), {*S*_e_ = 0.975; *S*_p_ = 0.975} (d = 0.035), {*S*_e_ = 0.950; *S*_p_ = 0.950} (d = 0.070), {*S*_e_ = 0.900; *S*_p_ = 0.900} (d = 0.140) and {*S*_e_ = 0.850; *S*_p_ = 0.850}, (d = 0.210) (Fig 2a).

### Whole-genome sequences

The GTEvaluator workflow was tested using 44 and 134 genomes of *Bacillus* spp., and *Salmonella enterica* serotypes respectively. For the two applications, no plasmid sequences were used but only chromosomic sequences both in contigs or closed genomes (annotated or not). Full genomes with conventional taxonomic information were collected across public databases (S1 Table).

For the first application, two groups were created: one including 22 genomes of *B*. *cereus* and the other with 22 genomes of *B*. *thuringiensis*. For the second application, 14 different groups were created corresponding to 14 different serotypes of *Salmonella enterica* subsp. *enterica* selected among the most frequently isolated in humans, animal and food [20, 21].

### Choice of genetic markers

Both couples of primers and probes can be used within GTEvaluator workflow. For the application on *Bacillus* spp., 22 couples of primers are included in the list input file (.txt) of the script ‘GTEvaluator_matrixMaker’. For the application on *Salmonella*, 17 couples of primers and 51 probes are compiled. These molecular markers are selected from a large selection of scientific articles published between 1998 and 2014 (S2 Table) [3–16].

The *Bacillus* spp. primers set were selected from studies focusing on toxins involved in gastrointestinal diarrhea and emetic syndromes: nonhaemolytic enterotoxin Nhe, Bce I, haemolysin BL (Hbl), hemolysin II (Hly II), cytotoxins K (Cyt-CK1 and CK2) and cereulide (Ces). Additional primers used to discriminate psychrotrophic and mesophilic strains were also included [3–10].

The genetic markers chosen for *Salmonella* application were selected from molecular studies focusing on high-throughput profiling of *S*. *enterica* serogroups and serotypes. Thirty-eight probes, designed by McQuiston *et al*. in 2011 [12] targeting the genes encoding the flagellar antigens H of the Kauffmann-White serotyping scheme [22], were chosen for their high discriminatory power among *S*. *enterica* serogroups. These probes are implemented in the xMAP^®^ *Salmonella* Serotyping Assay (Luminex, US) developed by the CDC [12, 13]. This assay consists of three separate tests that detect O and H antigens and some additional serotype-specific targets. This assay is able to identify 85% of most commonly encountered *Salmonella* serotypes in US [23].

We selected also 13 couples of primers and probes designed by Richmond *et al*. in 2011 and used in an innovating genotyping method that couple PCR and HPLC to identify *Salmonella* serotypes. This method was devised as part of a high-throughput mid-plexing analytical system to provide an efficient qualitative differential tool for the detection of several *Salmonella* serotypes [14].

Two genetic markers based on clustered regularly interspaced short palindromic repeats (CRISPR), described more recently by Fabre *et al*. in 2014, were also selected [11]. These molecular markers target specifically the causal agents of typhoid and paratyphoid fevers, and are used to differentiate the serotypes Typhi and Paratyphi A.

With regards to the emergent monophasic variant of Typhimurium (*S*. 4,[5],12: i: -) which is the third most common *S*. *enterica* serotype in Europe since 2011 [16], we selected several markers described by the literature for their ability to identify and differentiate *S*. Typhimurium from its variant 4,[5],12:i:- [15, 16].

## Results and discussion

### Plasmids are necessary to distinguish *B*. *cereus* from *B*. *thuringiensis*

Several molecular targets have been proposed to distinguish phenotypic traits in the *B*. *cereus* group like pathogenicity, toxicity or optimal growth temperature [3–10]. Recently, whole genome sequence data of a large number of *B*. *cereus* and *B*. *thuringiensis* strains have been made available, offering the opportunity to test the capacity of previously published genetic markers to discriminate *B*. *cereus* from *B*. *thuringiensis* [24, 25]. GTEvaluator was run on a set of 22 markers against 44 genomes (22 for the *B*. *cereus* group and for the 22 *B*. *thuringiensis* group). Among the genetic markers tested, HlyII and HB1 showed the best scores with *d* values of 0.71 and 0.39, respectively (Table 2). Nevertheless, these high *d* values resulted from both low sensitivities and specificities indicating the poor ability of these chromosomal markers to distinguish *B*. *cereus* from *B*. *thuringiensis*. This result is in agreement with the recent genomic studies demonstrating that *B*. *cereus* and *B*. *thuringiensis* are indistinguishable at the chromosomal level [24–25]. Actually, plasmids are the usual location of the more than 700 *cry* genes whose presence defines the species *B*. *thuringiensis* [26].

**Table 2:** Previously published targets presenting the lowest distances (*d*) calculated by GTEvaluator based on combinations of their respective sensitivity (S_e_) and specificity (S_p_). Uncertainties on sensitivity and specificity are presented for distances lower than 0.0140 and 0.0707. The bold characters indicate the promising values.

Group	Subgroup*	Number of genomes	Target	S_e_	S_p_	*d*	Probability d<0.014^#^	Probability d<0.0707^#^
*Bacillus*	*cereus*	22	HlyII	0.63	0.38	0.71	ND	ND
	*thuringiensis*	21	HB1	0.85	0.63	0.39	ND	ND
*Salmonella enterica* subsp. enterica	Agona	20	G-comp	1.00	0.81	0.18	<0.0001	0.0002
	Derby	1	G-comp	1.00	0.69	0.30	<0.0001	<0.0001
	Enteriditis	20	m-g_m	**1.00**	**1.00**	**0**	**0.18**	**0.78**
			SEN1383	**1.00**	**1.00**	**0**	**0.18**	**0.78**
			SEN1383_probe	**1.00**	**1.00**	**0**	**0.18**	**0.78**
	Hadar	2	EN-comp-1	1.00	1.00	0	0.03	0.21
			z10	1.00	1.00	0	0.03	0.21
	Infantis	3	SCH-2097-probe	1.00	0.97	0.02	0.03	0.22
			r	1.00	0.97	0.02	0.03	0.22
			SCH-2097	1.00	0.97	0.02	0.03	0.22
	Kentucky	11	z6	**1.00**	**1.00**	**0**	**0.10**	**0.58**
	Newport	20	e-h	1.00	0.97	0.02	0.13	0.69
	Panama	1	L-comp	1.00	1.00	0	0.02	0.13
	Paratyphi A	9	PA	**1.00**	**1.00**	**0**	**0.02**	**0.52**
			a-1	**1.00**	**1.00**	**0**	**0.02**	**0.52**
			a-2	**1.00**	**1.00**	**0**	**0.02**	**0.52**
	Saintpaul	3	e-h	1.00	0.84	0.15	<0.0001	<0.0001
	Typhi	17	TY	**1.00**	**1.00**	**0**	**0.16**	**0.73**
			d	**1.00**	**1.00**	**0**	**0.16**	**0.73**
			j	**1.00**	**1.00**	**0**	**0.16**	**0.73**
	Typhimurium	20	FliC	0.95	0.88	0.12	<0.0001	0.0048
	*S* 4,[5],12:i:-	4	FliC	1.00	0.78	0.21	ND	ND
	Virchow	3	SCH-2097-probe	1.00	0.97	0.02	0.0047	0.2156
			r	1.00	0.97	0.02	0.0047	0.2156
			SCH-2097	1.00	0.97	0.02	0.0047	0.2156

* *S* 4,[5],12:i:- corresponds to a monophasic variant of Typhimurium serotype,

^#^ ND stands for not determined.

With this first application including 22 *B*. *cereus* and 22 *B*. *thuringiensis* chromosomes, this negative-case example highlights the interest of GTEvaluator to rapidly eliminate non-discriminatory markers on a sound and quantitative basis.

### Evaluation of genetic markers for *Salmonella enterica* serotype discrimination

The classification of *Salmonella* isolates into serotypes is the official typing method used to monitor the spread of the foodborne pathogen and to trace back origin of contamination during epidemiological investigations. However, the conventional serotyping scheme of *Salmonella enterica* is an expensive and time consuming method. These drawbacks have led to the development of various molecular “serotyping” assays based on genetic markers. We applied GTEvaluator to evaluate the discriminatory values of 68 genetic markers selected from the literature [11–16] against genomes of the 14 most frequent serotypes encountered in human clinical cases [20, 21]. The GTEvaluator analysis was performed on a dataset of 134 genomes with a heterogeneous number of genomes per serotype (1 to 20) to highlight the importance of this parameter.

GTEvaluator has been run with these 68 markers and 134 genomes grouped according to serotypes. The full results (S_e_, S_p_, *d* and CI) are reported in S4 Table, while the first best hit for each serotype is displayed in Table 2.

Excepted for marker FliC based on *fliC* gene (S_e_ = 0.95; Table 2), all the genetic markers retrieved by GTEvaluator displayed an optimal sensitivity of 1.00 for the targeted serotypes, while the specificity was much more variable (ranging from 0.69 to1). The calculation of the distance which depends on both parameters (sensitivity and specificity) ranged from 0 to 0.3 for the 21 markers presented in the Table 2. The heterogeneity of the distance values was due to the variable specificity among markers (Table 2). Nevertheless, although distance was a good parameter to assess the quality of markers tested, it was not sufficient. The number of genomes on which the genetic markers were tested is also an important parameter to consider. Consequently, we associated Bayesian uncertainties to *d* values (see Methods) to take into account this issue. These results are presented in Table 2.

For six *Salmonella* serotypes: Enteritidis, Hadar, Kentucky, Panama, Paratyphi A, and Typhi, GTEvaluator recovered highly specific and sensitive markers (S_e_ = 1; S_p_ = 1; *d* = 0). However, the uncertainty (> 0.5) on *d* values suggested that only markers retrieved for Enteritidis, Kentucky, Paratyphi A and Typhi, can be considered as promising. For Hadar and Panama, although the *d* values were high, the low number of genomes on which sensitivity can be tested increased the uncertainty on *d*, making the performance of the marker on additional genomes questionable.

For the following pairs of serotypes: Agona and Derby, Newport and Saintpaul, Infantis and Virchow, GTEvaluator retrieved a pair-specific marker (Table 2). For Agona and Derby the G-comp marker [12] was selected with specificities values estimated at 0.81 and 0.69, and a high *d* values indicating that this marker has poor discriminative ability. This observation is not surprising because the G-comp probe was designed to recognize member of the flagellar G complex group which gather tens of different flagellar antigens [12, 22]. For Newport and Saintpaul, two serotypes sharing the same phase 1 flagellar e,h antigen, the e-h probe (designed to be specific of e,h single flagellar antigen) [12] was selected as suitable. This result emphasizes the relevance of GTEvaluator results and illustrates well how the number of genomes in subgroups impacted the *d* value and the uncertainty on it.

Concerning Infantis and Virchow, the same three markers have been selected: the SCH-2097 markers (probe and couple of primers) and the r probe [12, 14]. These results were coherent as SCH-2097 markers target the *rfb* gene cluster used to identify the serogroup C1 while the r probe targets the sequence coding for the r flagellar phase characterizing both *S*. Infantis and Virchow.

**Fig 3:**
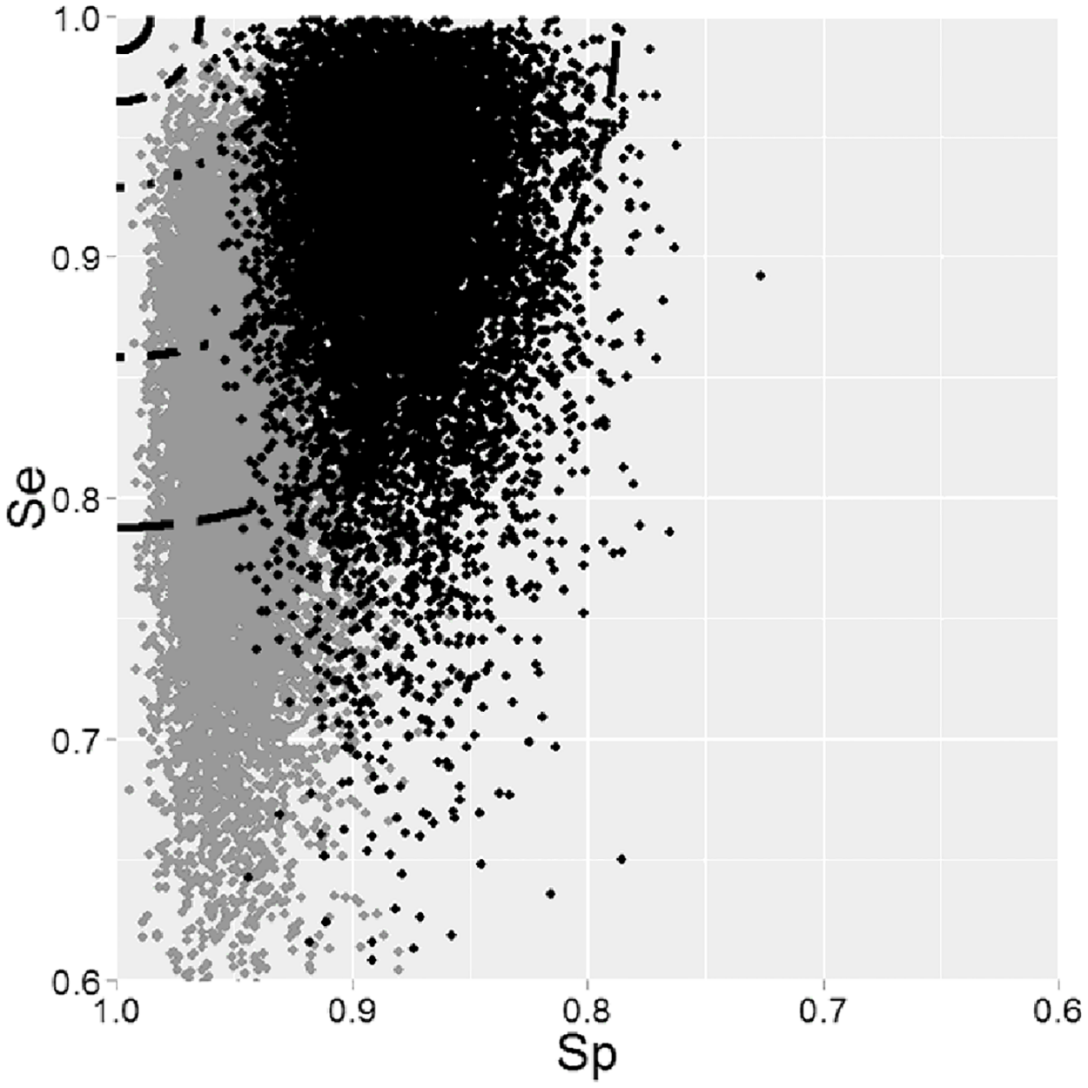
Graphical representation of the distances and uncertainties implemented in GTEvaluator for the genetic markers fliC and fljB for *Salmonella enterica* serotype Typhimurium. Confidence intervals of sensitivity and specificity of FliC (black) and FljB (grey) markers are represented according to their abilities to distinguish between 20 genomes of *S*. Typhimurium and 114 genomes of other serotypes of *Salmonella enterica*.

The FliC marker [15] was selected for the serotypes Typhimurium and its monophasic variant *S* 4,[5],12:i:-, but the *d* values were elevated, 0.12 and 0.21 respectively as a consequence of their low specificity (Table 2). Finally both FliC as FljB markers [15, 16], which target the *fliC* and *fljB* gene clusters encoding the flagellar H antigens, had the more appropriated hit for Typhimurium and its variant (S3 Table). Nevertheless they cannot both be used to distinguish these two serotypes from the others. With high sensitivities and specificities, these two markers could appear as suitable, yet it can be difficult to rank them due to the overlap of their interval confidence zones (Fig 3 and S3 Table). More genomes would help to distinguish their predictive performance for the Typhimurium serotype.

The couples of primers published by Fabre *et al*., in 2014 [11] to distinguish *Salmonella* Typhi from Paratyphi A, were identified by GTEvaluator as highly discriminant. This result confirms that CRISPR sequences are appropriate targets for the PCR assay developed to identify these two serotypes.

The results obtained with GTEvaluator on the 68 genetic markers selected for discriminating different *Salmonella enterica* serotypes were in accordance with most of the published data. Indeed, except for the CRISPR sequences that are able to discriminate Paratyphi A and Typhi [11], the other molecular markers cannot be used in isolation. The discrimination of the other serotypes can only be possible by using these genetic markers in combination.

It is also important to emphasize that the size of the genome dataset of a given subgroup is critical because markers having poor statistical performance in a small dataset (i.e. high distance values and high uncertainties) may finally be appropriate with a larger genome dataset. It must also be noted that the diversity of genomes within polyphyletic serotypes must be taken into account. In the present dataset this parameter did not influenced the results because the molecular markers analyzed were selected from studies focusing on high-throughput profiling of *S*. *enterica* serogroups essentially targeting genes encoding the somatic and flagellar antigens of the Kauffmann-White serotyping scheme [22]. For example, Newport e-h markers [12] selected display a good performance even though the 20 Newport genomes correspond to five different MLST profiles (data not shown).

## Concluding remarks

We have presented two situations (*Bacillus* and *Salmonella*) in which GTEvaluator was able to rank genetic markers toward a genomes dataset and found that it is an appropriate tool to accurately evaluate the most suitable genetic markers among a predefined list of markers. The GTEvaluator results were further evaluated through a statistical approach to measure the relevance of markers. The results obtained with these two situations allowed us to discuss the parameters (specificity, sensibility, distance value and uncertainties of distance defined by a Bayesian stochastic approach) used to rank the markers. The size of genome dataset, the diversity of the genomes included in the dataset and the selection of plasmid and/or chromosomal sequences were also mentioned in the discussion.

Finally the results obtained on the “*Salmonella* dataset” showed that some serotype (Enteritidis, Kentucky, Paratyphi A, and Typhi) could be confidently assigned with one genetic marker, while others would need further developments.

Based on WGS, single-nucleotide polymorphism (SNP)-analysis, whole genome- or core genome- Multi Locus Sequence Typing (MLST) are efficient and can be considered as ultimate typing methods [27, 28]. However, beside WGS, typing or detection methods using polymerase chain reaction (PCR)-based assays present numerous advantages in term of timeliness, versatility or cost. In the future it is likely that microfluidic or room temperature PCR devices will continue to be developed and will find novel applications in environmental, medical or food sectors [29]. Consequently, there will be a continuous demand on the capacity to design specific and sensitive molecular markers. In that perspective, the increasing amount of genome sequences provides a data goldmine that should be exploited.

With this in mind, we conceived GTEvaluator as a post-sequencing tool, taking advantage of available genomic data to provide a sound statistical estimation of the performance of genomic markers.

Thus the main purpose of GTEvaluator is to allow the evaluation of genetic markers that could be used in alternative detection or typing methods, not necessarily requiring sequencing of whole genome or even isolation of microorganisms.

In a context where the availability of whole-genome data is growing strongly, the possibility offered by GTEvaluator to rapidly screen *in silico* for group-specific genetic markers is extremely useful. In its present state, GTEvaluator produces a single matrix resuming the ‘presence’ (100% of sequence homology) and ‘absence’ of molecular targets across genomes (S3 Table). It is of course fully possible to use this output to design combination of genetic markers to develop more elaborated selecting strategies. The GTEvaluator tool has been developed to evaluate the performance of markers independently of their design. Given the wealth of genomic data produced it is now important to develop bioinformatics tools associated to robust statistical functions, to retrieve original markers which can be used for various typing strategies. In that last perspective, we are currently developing such a bioinformatics pipeline to extract from genome subgroups, sensitive and specific genetic elements.

## Supporting information

S1 Table List of genomes used in the present study focusing on *Bacillus* spp. and *Salmonella enterica* serotypes. The name of organism, the GenBank accession number, whole genome sequencing (WGS) project codification and amount of scaffolds are specified.
(XLSX)

S2 Table List of genetic markers (primers and probes) use in the present study focusing on *Bacillus* spp. and *Salmonella enterica* serotypes. * stands for corrected sequences.
(XLSX)

S3 Table Sensitivity (S_e_) and specificity (S_p_) of genetic markers across genomes of *Bacillus* spp. and *Salmonella* enterica serotypes. CI and POS stand for confidence interval and graphical positions, respectively.
(XLSX)

S4 Table Matrices resuming the ‘presence’ (i.e. ‘1’) and ‘absence’ (i.e. ‘0’) of genetic markers across genomes of *Bacillus* spp. and *Salmonella enterica* serotypes. The references of the genetic markers are listed in S2 Table.
(XLSX)

## Abbreviations

CI: confidence interval
*d*: distance
GTEvaluator: Genome Target Selector
S_e_: sensitivity
S_p_: specificity
x_ij_: genetic marker
